# Proline-conditioning and chemically-programmed ice nucleation protects spheroids during cryopreservation†

**DOI:** 10.1039/d3cc02252h

**Published:** 2023-07-04

**Authors:** Yanan Gao, Akalabya Bissoyi, Nina L. H. Kinney, Thomas F. Whale, Qiongyu Guo, Matthew I. Gibson

**Affiliations:** a Department of Chemistry, University of Warwick Coventry CV4 7AL UK m.i.gibson@warwick.ac.uk guoqy@sustech.edu.cn; b Division of Biomedical Sciences, Warwick Medical School, University of Warwick Coventry CV4 7AL UK; c Department of Biomedical Engineering, Southern University of Science and Technology Shenzhen Guangdong 518055 China

## Abstract

Spheroids mimic 3-D tissue niches better than standard cell cultures. Cryopreserving spheroids, however, remains challenging as conventional cryoprotectants do not mitigate all damage mechanisms. Here chemically-programmed extracellular ice nucleation is used to prevent supercooling, alongside proline pre-conditioning, which are found to synergystically improve post-thaw recovery of spheroids. This validates the need to identify compounds and materials to address both biochemical and biophysical damage pathways beyond standard cryoprotectants.

## Abstract 2

Addressing both biochemical and biophysical mechanisms of cryo-damage through proline preconditioning and induced ice nucleation rescues spheroids from cryopreservation damage.

## Graphical abstract

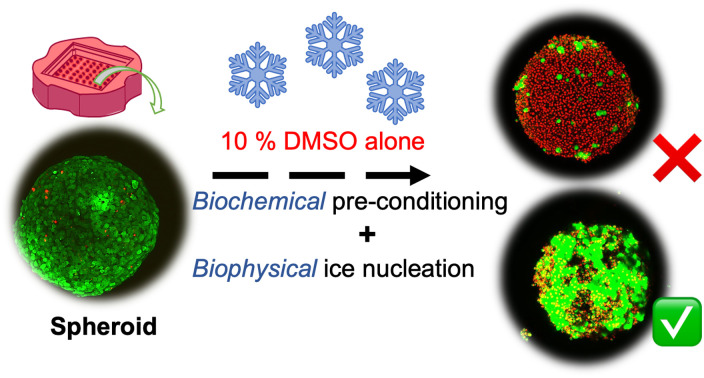

Cell culture is a crucial tool to explore biochemical pathways, to discover new drugs and to produce biologics. Continuous cell culture leads to phenotype drift,^1^ and is resource intensive, necessitating cryopreservation to allow banking, and distribution. Cryopreservation is successful for cells in suspension but 2-D monolayers and 3-D models (such as spheroids and organoids^2,3^) are more challenging to store. 3-D Models are more predictive of *in vivo* fate (such as toxicology^4,5^). In 2022 the US FDA removed some requirements for animal testing prior to human studies, where advanced cellular models are available.^6^ There is a clear need to develop and discover freezing strategies to protect 3-D tissue models to allow their wider uptake and to improve reproducibility.^5^

Conventional cryopreservation methods use ∼10% DMSO, but there is growing interest in a chemistry-driven approach to discovering compounds and materials which mitigate other mechanisms of cryopreservation-induced damage,^7^ including ice growth^8^ and delayed onset apoptosis.^9^ Polyampholytes can protect 2-D and 3-D cellular models,^10–13^ potentially through membrane stabilisation and cellular dehydration. During cryopreservation, water tends to supercool, meaning nucleation of ice does not occur until ∼−20 °C in microliter droplets^14^ and −15 °C in multiwell plates.^15,16^ Induced nucleation within 96 well plates is known to increase recovery of adherent (2D) cell monolayers post-thaw.^15,16^ Induced nucleation at warmer temperatures reduces intracellular ice formation (IIF) and leads to less ice propagation at cell–cell contacts^17^ which is particularly relevant for spheroids.

There are few useful ice nucleators for cellular cryopreservation. Ice nucleating proteins from bacteria are not isolated pure, only as lyophilized powders.^18^ Minerals such as feldspar^19,20^ can nucleate but require specialised devices to segregate the insoluble powders from the cells.^21^ Physical nucleation requires external stimuli, at a precise point in the freezing process, requiring thermal monitoring. Soluble polysaccharides extracted from pollen have emerged as potent ice nucleators^22^ and we have shown they improved post-thaw recovery of cryopreserved spheroids by inducing nucleation at a pre-determined and reproducible temperature.^23^

In addition to the physical modes of damage/protection, a ‘medicinal chemistry’ approach to cryopreservation, targeting specific deleterious pathways, can mitigate cold damage. ROCK^24,25^ (Rho-associated kinase) and caspase^26,27^ inhibitors improve post-thaw yield. l-Proline, as well as being a protective osmolyte, can promote stress tolerance in several species.^28,29^ Application of proline to cell monolayers pre-conditions them for freezing and can improve cell recovery^30,31^ although the mechanism and cell scope of this is not understood.

Here we explore the synergy of proline preconditioning (biochemical) and induced extracellular nucleation (biophysical) for the cryopreservation of spheroids. Each approach alone gives benefit, but together lead to substantial improvements in cell recovery. There is evidence for reduced reactive oxygen species and actin depolymerization, as a consequence of this approach, highlighting the importance of discovering new cryoprotectants for emerging cellular technologies.

A549 adenocarcinomic human alveolar basal epithelial cells, were cultured and assembled into spheroids,.^10,11,32^ Spheroids were prepared using an agar-mould^33,34^ to ensure homogeneity, seeded with 8000 cells per spheroid and allowed to grow for 5 days before use, Fig. 1. It is important to note that this is a relatively large and dense spheroid, chosen to be a challenging cryopreservation target.

**Fig. 1:**
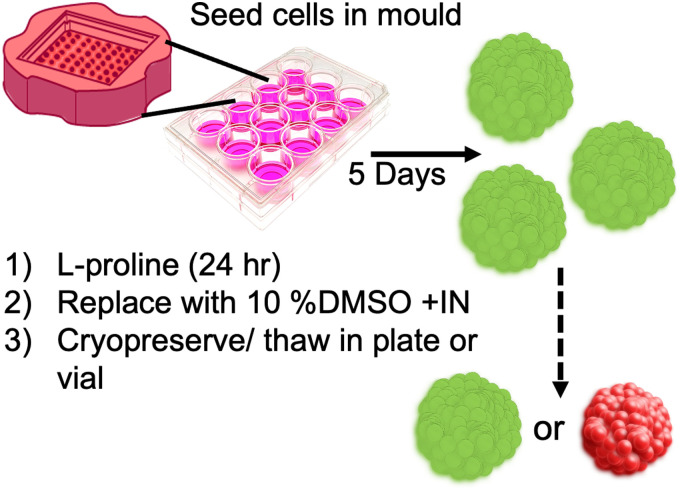
Spheroid cryopreservation process. l-Proline applied for pre-incubation before cryopreservation. +IN Indicates addition of ice nucleator from *Carpinus betulus* pollen. Green/red indicates live/dead spheroids.

To evaluate the role of proline-pre-conditioning, the spheroids were exposed to 50–300 mM of proline for 24 hours, which was then removed (no proline remained in the cryopreservation medium) and replaced with 10% DMSO in cell culture medium and cryopreserved. Proline has a unique cryoprotective mechanism,^30,35^ beyond its impact on osmolarity, which is not seen for other amino acids, although some mitigate ice recrystallisation.^36^ The spheroids were thawed, and allowed to recover for 24 hours (to prevent false positives^32^). The recovery rate of the spheroids was then measured by ATP content, Fig. S3B (ESI†). In this initial screening, spheroids were cryopreserved in their moulds, for convenience, not to maximise the absolute post-thaw recovery. (Due to the agar around the spheroids additional stress is placed during freeze/thaw.) As expected, within moulds, 10% DMSO gave a low recovery of ∼5%. Spheroids which were pre-treated with 300 mM proline, however, showed a significant increase in recovery to 10%, indicating the proline can assist spheroid recovery, as seen for A549 and Neuro-2a monolayers.^30,31^ No cytotoxicity was observed at these proline concentrations (ESI†).

With the benefit of proline observed, chemically-induced ice nucleation was explored, Fig. 2(A). An additive to program the nucleation temperature is practically far simpler than physically induced nucleation using electrofreezing^37^ or mechanical shock.^38^ Soluble ice nucleating macromolecules were extracted from *Carpinus betulus* pollen, following reported procedures.^16,23^ These extracellular macromolecules increase the nucleation temperature of water from −15 °C to as high as −6 °C in microwell plates.^16^ Fig. S8 (ESI†) illustrates the ice nucleation activity of the pollen washing water in 10% DMSO in microliter droplets, demonstrating it can reduce super-cooling and hence less intracellular ice formation.^16,17,21^Fig. 2(A) shows the results of cryopreservation using DMSO alone, and with either or both of proline pre-incubation and induced nucleation (+IN). [Note, it was observed that the volume of liquid was crucial to maximise recovery and a lower volume was used here (200 μL) compared to the original screening (Fig. S3, ESI†), using 1 mL]. In all cases induced ice nucleation increased recovery, with the highest (∼40%) achieved when proline and induced nucleation were combined. Reduced [DMSO] concentration impaired post-thaw outcomes. Confocal microscopy confirmed that using DMSO alone, some spheroids lost cohesion. But with induced nucleation and proline addition, the spheroids were partially intact, albeit with damage compared to unfrozen controls.

**Fig. 2:**
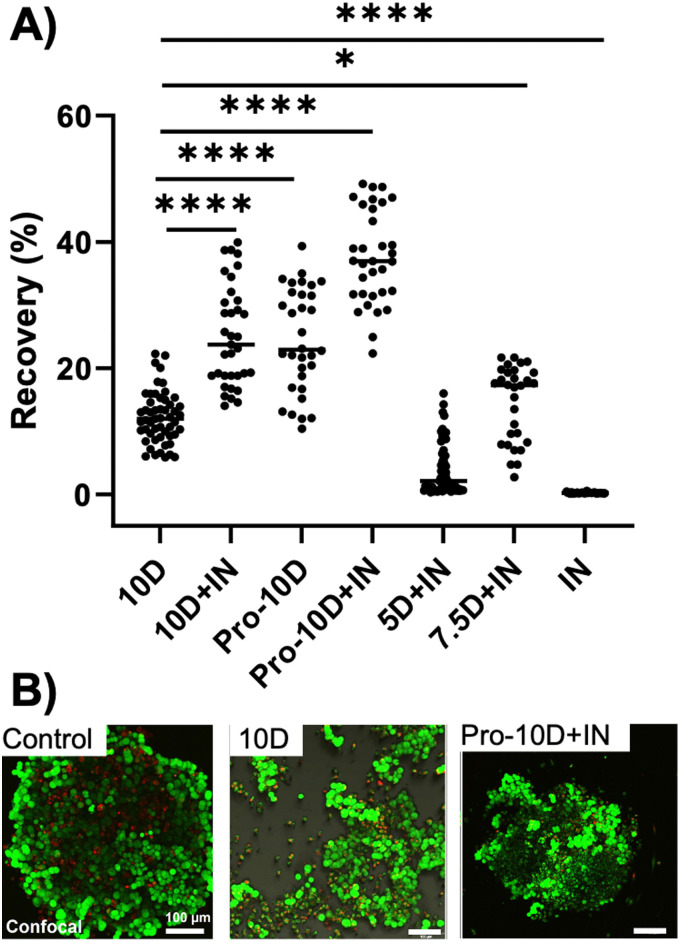
Post-thaw 24 h recovery of cryopreserved A549 spheroids in agarose moulds, determined by total ATP content. Seeding density 8000 cells per spheroid. (A) Recovery with/-out 300 mM proline pre-incubation (Pro) and cryopreserved in agarose moulds with/-out active ice nucleation (+/−IN) and indicated DMSO concentration, 200 μL total volume/well; (B) confocal imaging before and after thawing in 10% DMSO (10D) compared to 300 mM l-proline (Pro) and ice-nucleation(+IN). Green (live cells, calcein-AM), red (dead cells, EthD-III). Scale bar: 100 μm. *P < 0.05; **P < 0.01; ***p < 0.001; ****P < 0.0001.

To increase recovery, spheroids were transferred from moulds to u-bottom 96-well plates, which have been used for spheroid freezing with induced nucleation.^23^ Using identical freezing conditions as above, significantly higher recoveries were observed with 10% DMSO giving 40%, which is increased to >70% by the synergy of proline and nucleation. Our approach is distinct from standard formulation methods to optimise solvent cryoprotectants (such as DMSO) and osmolytes (*e.g.* trehalose^39^). To probe the impact of these strategies on spheroid function A549 and HepG2 spheroids were investigated using confocal microscopy. Live (green)/dead (red) staining was used showing that for both A549 (Fig. 3(A)) and HepG2 (Fig. 3(B)) the proportion of live (green) cells was larger upon proline and nucleation treatment, compared to 10% DMSO alone. Furthermore, a reactive oxygen species assay (stained green) showed that in 10% DMSO alone there was more ROS than in the controls or the proline/nucleated sample. Standard cryopreservation methods do not address ROS and other apoptotic stress pathways, although specific ROS inhibitors have been reported.^25^ We cannot rule out a ROS suppression mechanism, compared to simply more healthy cells, which would also show less ROS. The exact mechanism of how proline protects cells is not clear, as several pathways are modulated.^31^ F-actin staining (ESI†) showed rescue of F-actin polymerization relative to DMSO alone, which is tentatively attributed to reduced intracellular ice formation. This has also been reported for polyampholytes which may aid cellular dehydration by other mechanisms.^10,11,40^ Overall HepG2 cell recovery was lower than for A549 but this was not optimised here, but used to show the proline/nucleation combination was broadly applicable. HepG2 model cytotoxicity tests against doxorubicin (ESI†) confirmed improved functional performance compared to 10% DMSO alone. It should be noted the recovery level here is lower than for other spheroids with nucleation.^23^ The agar-templated spheroids here are more dense (400 μm *vs.* 800 μm for equal cell seeding density), which might lead to more inter cellular ice propagation or slow DMSO removal post-thaw, which will be investigated in the future.

**Fig. 3:**
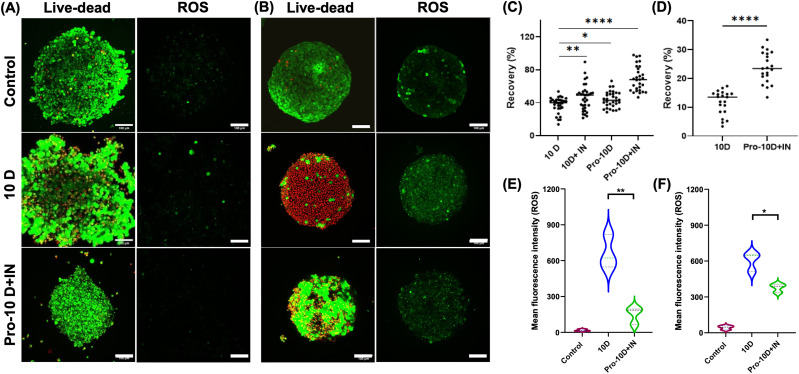
24 h Post-thaw recovery of cryopreserved spheroids in U-96 well plates. Confocal images of (A) A549 spheroids and (B) HepG2 spheroids. Live = green, dead = red. ROS (reactive oxygen species) staining, ROS = green. Control is non-frozen; (C) recovery of A549 spheroids and (D) HepG2 with indicated cryoprotectants. Mean fluorescence intensity of ROS of A549 spheroids (*n* = 3) (E) and HepG2 spheroids (F) before and post-thaw 24 h. For all data, Pro indicates 24 h pre-incubation with 300 mM proline; +IN indicates nucleator added and 10D = 10% DMSO. Scale bar: 100 μm. *P < 0.05; **P < 0.01; ***p < 0.001; ****P < 0.0001.

In conclusion, we have demonstrated the synergy between mitigating both biochemical and biophysical modes of damage during spheroid cryopreservation. Initial screening of spheroids in agar moulds confirmed that 24 hours pre-incubation with proline (which was removed prior to freezing) increased post-thaw recovery levels of A549 spheroids. Addition of soluble ice nucleators (from pollen) to prevent super-cooling and reduce intracellular ice formation, also increased recovery. Combining these two strategies in microwell-plate based freezing enabled an overall increase in spheroid recovery from 40 to 70%, which was shown to be associated with reduced reactive oxygen species and increased F-actin polymerization. HepG2 spheroids cryopreserved by this strategy showed improved function in a toxicology assay. Initial observations that spheroid density impairs function were also made. This work shows that the chemistry-led discovery and application of specific biochemical inhibitors and extracellular nucleators can advance the banking and deployment of 3D models for basic and applied research.

MG, YG and TW devised the experiments. YG and AB undertook the cell culture experimental work. NLHK performed ice nucleation experiments. MG and QG supervised the project. All authors contributed to data analysis and manuscript writing.

This project has received funding from the European Research Council under the European Union's Horizon 2020 research and innovation program grant agreements 866056 and 899872. MIG thanks the Royal Society for an Industry Fellowship (191037) with Cytiva. AB thanks BBSRC/UoW for a Flexible Talent Mobility Award (BB/W510907/1). NLHK thanks NERC (NE/S007350/1) and YG thanks SUSTECH/UoW for PhD scholarships. TFW thanks the Leverhulme Trust/UoW for a Fellowship (ECF-2018-127). Q. G. thanks the National Natural Science Foundation of China (81971764) and the Innovation Committee of Shenzhen Municipality (JCYJ 20200109141638004). For the purpose of open access, the author has applied a Creative Commons Attribution (CC BY) license to any author-accepted manuscript version arising from this submission.

## Conflicts of interest

TFW, NLHK and MIG are named inventors of a patent application relating to this work.

## Supplementary Material

CC-059-D3CC02252H-s001
