# Supplementary material for: Proline-conditioning and chemically-programmed ice nucleation protects spheroids during cryopreservation
Source: Chem Commun (Camb). 2023 Jul 4;59(59):9086–9. doi: 10.1039/d3cc02252h (PMC10358716; doi:10.1039/d3cc02252h)
Supplement: CC-059-D3CC02252H-s001 [file CC-059-D3CC02252H-s001.pdf]

Electronic Supplementary Material (ESI) for Chemical Communications.
This journal is © The Royal Society of Chemistry 2023

Supporting Information For:

Proline-Conditioning and Chemically-Programmed Ice Nucleation Protects Spheroids During Cryopreservation

Yanan Gao,[a,c] Akalabya Bissoyi,[b] Nina L. H. Kinney,[a] Qiongyu Guo,[c*] Thomas F. Whale [a*] and Matthew I. Gibson [a,b*]

[a] Department of Chemistry, University of Warwick, Gibbet Hill Road, Coventry, CV4 7AL, UK;

[b] Division of Biomedical Sciences, Warwick Medical School, University of Warwick, Gibbet Hill Road, Coventry, CV4 7AL, UK;

[c] Department of Biomedical Engineering, Southern University of Science and Technology, Shenzhen, Guangdong 518055, China.

Experimental Section

All materials were used as supplied unless otherwise stated.

Cells: Human Caucasian lung carcinoma cells (A549), Human Hepatocellular carcinoma (HepG2, Loughborough, UK, ECACC85011430).

Materials: Dulbecco's phosphate buffered saline (DPBS, Thermo Fisher Scientific,D8537), Ham's F-12K (Kaighn's) Medium (Gibco, Paisley, UK), non-US origin fetal bovine serum (FBS) (Sigma Aldrich, Dorset, UK, F7524) and PSA (HyClone, Cramlington, UK). 0.25% trypsin plus 1 mM EDTA (Gibco, 25200072), Minimum Essential Medium Eagle medium (MEM, M4655), agarose Bio Reagent for molecular biology (Sigma, A9539), sterile saline [0.9% (w/v) NaCl], CellTiter-Glo® 3D Cell Viability Assay (Promega, G9682, USA), Hoechst 33342 (Life Tech., CA, USA). *Carpinus betulus* (hornbeam) pollen (CARB.0116, Pharmallerga, Lisov, Czech Republic). CoolCell™ LX cell freezing vial container (CLS432001), Corning®XT CoolSink®96F thermoconductive plate (Merck, Gillingham, UK, CLS432070). Live-Dead Viability/Cytotoxicity kit (2326049), Invitrogen ActinGreenTM 488 ReadyProbeTM reagent (R37110) and cryovials were all purchased from Thermo Fisher (Loughborough, UK). 10% dimethyl sulfoxide hybrid-max, sterile-filered (D2650), Triton X-100 and 0.4 % trypan blue (T8154), L-proline (Irvine, UK), doxorubicin hydrochloride 98% (DOX, 860360) was purchased from Sigma Aldrich Co Ltd.

Equipment: Incubator (Haier Biomedical, HCP-168, UK), centrifuge for 15 mL tubes (VF4G003085), Invitrogen ™ Countess™ 3 FL Automated cell counter, BioTek Synergy HT microplate reader, an inverted microscope (Olympus, Southend-on-Sea, UK), VIA freeze (Asymptote Ltd, Cambridge, UK).

Methods

Cell culture

Human Caucasian lung carcinoma cells (A549) from the European Collection of Authenticated Cell Cultures (ECACC) (Salisbury, UK) and were seeded at density of 1.5×10^6 cells per T175 cell culture Nunc flasks (Corning, NY) using 40 mL of Ham's F-12K (Kaighn's) Medium (Gibco, Paisley, UK) supplement with 10% fetal bovine serum (FBS) (Sigma Aldrich, Dorset, UK) and 1% PSA (HyClone, Cramlington, UK). Cells were kept in an incubator in a humidified atmosphere containing 5% CO_2 at 37 °C. After over 90% confluency, the subconfluent in T175 was washed with DPBS (Thermo Fisher Scientific), using 10 mL of 0.25% trypsin plus 1 mM EDTA (Gibco) dissociation for 5 min. 10 mL total media was subsequently added to end enzymatic reaction. The solution was then centrifuged at 2000 rpm for 5 min. The cells were grown at 37 °C with 5% CO_2 in air. Human Hepatocellular carcinoma (HepG2) cells were cultured using Dulbecco's minimal essential medium (DMEM) supplemented with 10% fetal bovine serum, penicillin (100 units/ml), and streptomycin (100 µg/mL) at 37 °C with 5% CO_2.

Fabrication of spheroids

3D Petri Dishes®

1 g of pure agarose powder (Sigma) was measured and placed in a dry 100 mL autoclave-safe glass bottle for autoclave 30 minutes. Swirling the bottle after 50 mL of sterile saline [0.9% (w/v) NaCl] was added to the bottle containing agarose power in a biosafety cabinet. The solution was then heated using a microwave oven to boil and completely dissolve the power. Pipetting 500 µL of agarose solution in 3D Petri Dishes® for 10 min for gelation. The agarose micro-moulds were subsequently removed from the 3D Petri Dishes before transferring them in a 12-well plate and equilibrating with cell culture medium for 30 minutes in an incubator. Extracting all culture medium before seeding cells in a micro-mould and 190 µL of A549 cells

suspension was placed in each chamber containing 648,000 cells (~8000 cells/spheroid). Plates left to set for at least 10 min and then adding 2.5 mL medium to each well outside of the mould. Spheroids were cultured for up to 7 days. A549 cells were grown in F-12 K medium and placed in an incubator and renew medium every third day. HepG2 cells were cultivated using MEM supplemented with 10% (v/v) FBS, 1% antibiotic-antimycotic solution 100x, 1% MEM Non-Essential Amino acid solution 100X in the same formation method as A549 spheroids. An inverted microscope (Olympus, CX41, Southend-on-Sea, UK) were applied to monitor spheroid formation and spheroids images analysis used ImageJ software v1.52.

Slow freezing spheroids

a. Three ways for cryopreservation of spheroids:

A) Spheroids were frozen directly in agar micro-moulds with 1 mL cryoprotectant solution in 12-well plates in a controlled-rate freezer (VIA freezer™), -1 °C/ min until it reached -80 °C, then plates were transferred to -80 °C freezer for 24 hours.

B) Spheroids were frozen directly in agar micro-moulds, however, we optimised the method with 200 µl cryoprotectant solution, 12-well plates were placed on a CoolCell® MP plate (BioCision, LLC, Larkspur, CA) and transferred to a -80 °C freezer for 24 hours.

C) To further improve the recovery, spheroids were transferred to U-bottom 96-well plates, one spheroid with 50 µl cryoprotectant solution each well and then plates were placed on CoolCell® MP plates and were stored in a -80 °C freezer directly.

Different concentrations of solutes dissolved in the cell culture medium containing indicated concentrations of DMSO with or without pollen washing water (PWW) as an ice nucleation agent (+ IN) in 12-well plates (Corning, NY). 10% DMSO group as the control experiment.

b. Thawing methods

A) B) After 24 h storage at -80 °C, the 12-well plates were thawed by adding 1 mL pre-warmed complete cell culture medium, plates were placed in the incubator for 5 min to fully thaw and replaced with 1 mL complete cell culture media for the other 24 hours incubation.

C) Complete cell culture medium warmed to 37 °C was added into U-96 well plates, 100 µl/well. Plates were placed in a humidified atmosphere for 5 min to thaw and the cell culture media replaced with 100 µl complete cell culture media before moving back to the incubator for 24 h culture.

The spheroids were transferred individually to a white 96 well plate for the luminescence assay to determine the recovery and the viability before and after thawing. Unless stated otherwise, the spheroids were 8000 cells for all tests. Images of before/after freezing were captured by an Olympus CX41 microscope.

Evaluation of cell viability morphology

Before freezing and post-thaw, we used a CellTiter-Glo® 3D Cell Viability Assay (Promega, G9682, USA), which is a homogeneous method to determine cell viability in 3D cell culture based on quantitation of the ATP present. ATP is the marker for the present of metabolically active cells. The kit is a involves with more robust lytic capacity and is designed for microtissues sample. We picked one spheroid with 100 µL media to each well of a 96-well white plate, added 20 µl/well, mixed by a BioTek Synergy HT microplate reader shaking for 5 min to lyse cells and stabilize ATP. The plate was placed in dark for 25 min at room temperature. Subsequently, a luminescence BioTek Synergy HT microplate reader was used to analyse the viable cells.

The LIVE/DEAD® Viability/Cytotoxicity Assay Kit was used to observe apoptotic cell death and morphology of spheroids. It provides a two-colour fluorescence cell viability assay that is based on the simultaneous determination of live and dead cells with two probes that

measure recognized parameters of cell viability- intracellular esterase activity and plasma membrane integrity. For this assay, remove the media from each well, the spheroids were kept in the mould and washed with DPBS for two times. An approximately 2 μM of calcein AM (5 μL) and 4 μM of ethidium homodimer-1 (20 L) were added to 10 mL of sterile D-PBS in a 15 mL tube with 33 μM Hoechst 33342 (Life Technologies, Carlsbad, CA), vertexing to ensure thorough mixing. Then, 200 μL solution was added in each mould and incubated at room temperature for 1 h, spheroids were then transferred to confocal dishes and imaged using a FV3000 confocal laser-scanning microscope (Olympus, Tokyo, Japan). The polyanionic dye calcein AM retained within live cells, showing an intense uniform green florescence in live cells at ex/em ~495 nm/ ~515 nm. EthD-1 entered cells with damaged membranes, binding to nucleic acids and producing a bright red fluorescence in dead cells at ex/em ~495 nm/ ~635 nm.

Cytoskeleton

Samples (8000 cells/spheroid) were fixed with 4% paraformaldehyde (PFA) in PBS for 20 minutes at room temperature. Permeabilize the spheroids with 0.1% Triton X-100 in PBS for 15 minutes, washing twice with DPBS and then spheroids were incubated with primary antibody for cytoskeleton dye (Incitrogen ActinGreen ™,488 ReadyProbe reagent overnight at 4 degrees in the fridge. The samples were washed once with DPBS before imaging with a FV3000 confocal laser-scanning microscope (Olympus, Tokyo, Japan), ex/em ~ 499/520 nm. F-actin mean fluorescent intensity of the images were analysed by using ImageJ v1.52. The spheroids cultured for 6 days as a control and spheroids cryopreserved with 10D or Pro-10D+IN group were analysed. The percentage of fluorescence intensity of F-actin is equal to the value of each group divided by the mean of the control group.

Reactive Oxygen Species (ROS) assay

ROS detection reagent (Invitrogen, D399) was applied for spheroids imaging. Samples of before and after-freezing were washed with a DPBS buffer before incubating with 200 μl dichlorodihydrofluorescein diacetate (DCFDA) solution (20 μl of 7.5 mM diluted stock in PBS)/ well in U-96 well plates for 30 minutes. The spheroids were transferred to confocal dishes and captured using the FV3000 confocal laser-scanning microscope (Olympus, Tokyo, Japan) with fluorescence excitation and emission in ~495/515 nm. ImageJ v1.52 was used to analyse the mean fluorescent intensity of ROS. Three images of each group were measured, the value of mean fluorescence was calculated using mean fluorescence of the spheroids minus that of background.

Ice Nucleation Assay

Ice nucleation temperatures of 5 μl droplets were measured using a custom-built droplet freezing assay. The pollen washing water (PWW) solution for nucleation measurements was prepared by adding 0.04 g of Carpinus betulus pollen (Pharmallerga®) to 2 ml of a 10% DMSO solution. The pollen suspension was refrigerated overnight before filtering through a 0.2 μm syringe filter into a clean glass vial. For the ice nucleation measurements, twenty 5 μl droplets of the filtered PWW were pipetted onto a 22 mm diameter Hampton Research HR3-231 siliconized glass slide using a Sartorius Picus® electronic micropipette. This slide was placed onto the cold stage, which was used to reduce droplet temperature at a rate of 2 °C/min. Droplet freezing was monitored using a digital camera, allowing the fraction of droplets frozen as a function of temperature to be determined.

Statistical Analysis: Origin 2022b, ImageJ v1.52 and GraphPad Prism 9 software were used to analyse the data. To determine significance between the means of two groups, an unpaired two-sided T-test was conducted using GraphPad Prism 9 Software.

Additional Data

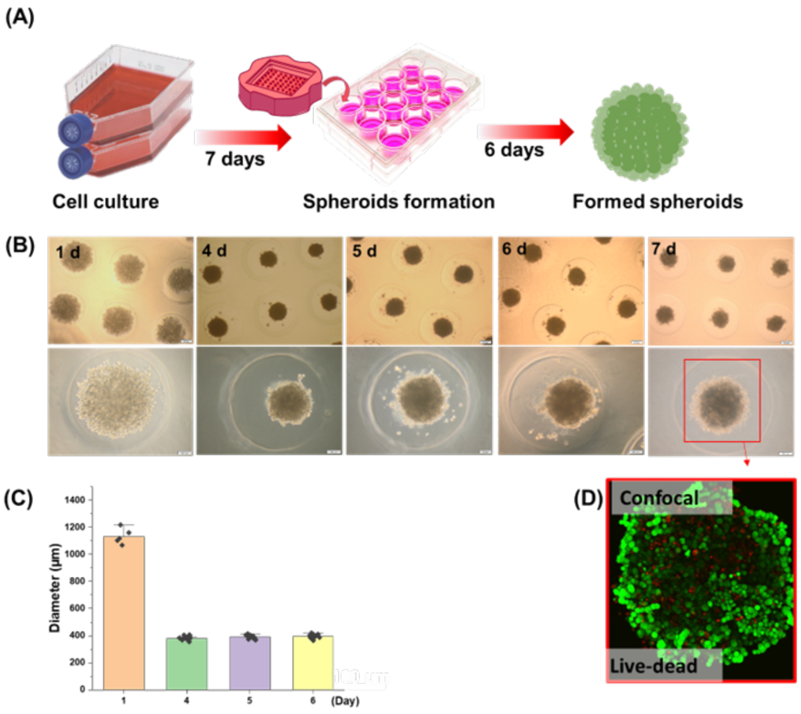

Figure S1. Formation and evaluation of A549 spheroids. (A) Schematic of fabrication of spheroids. (B) The formation of spheroids (8000 cells/ spheroid) in micro-well of agarose moulds, grown for 7 days. (C) Diameter of spheroids (8000 cells) from day 1 to day 6. (D) Confocal image of a A549 spheroid at day 7. Scale bar: 200 μm (top), 100 μm (lower).

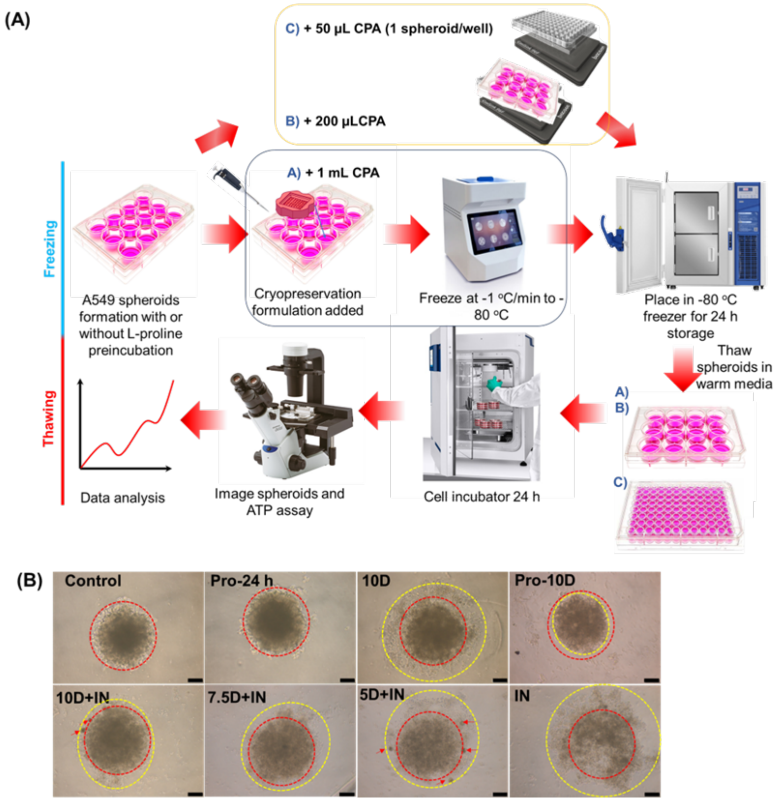

Figure S2. Cryopreservation of A549 spheroids. (A) Schematic of cryopreservation processes of three methods. (B) Spheroids before and cryopreserved using method A) with 10% DMSO (10D) supplementation with or without 300 mM L-proline (Pro) and ice-nucleation agent (+IN) under bright field (red circle dash line represents the area of spheroids before freezing; yellow circle dash line represents the area of spheroids post-thaw 24 h), 7.5D: 7.5% DMSO, 5D: 5% DMSO. Scale bar: 100 µm (B).

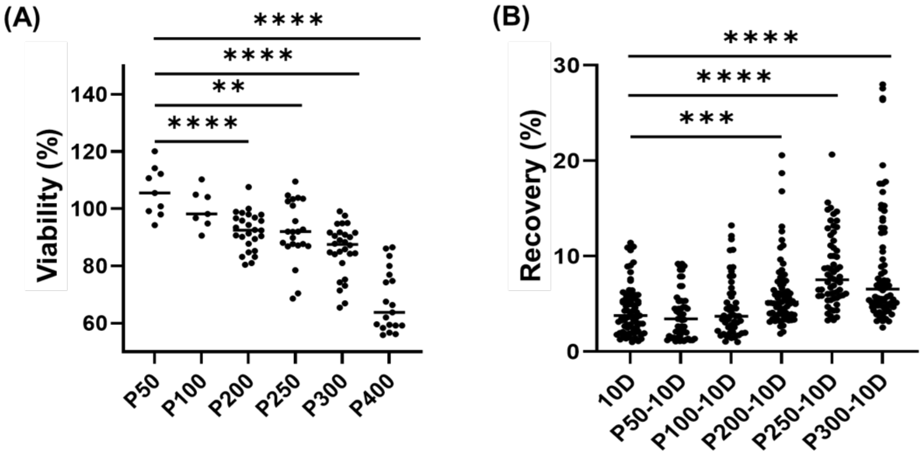

Figure S3. L-proline pre-incubation improved the recovery of A549 spheroids after cryopreservation. (A) Percentage of viability of spheroids incubation with various concentrations of proline (P50-P400: 50-400 mM), incubation for 24 hours, the middle black line showed the mean of each group. (B) Recovery as a function of L-proline concentration (Pro/mM) pre-incubation for 24 hr and cryopreserved with 1 mL of 10% DMSO in agarose moulds in 12-well plates; ** P<0.01, **** P<0.0001. CellTiter-Glo® 3D Cell Viability assay was used to determine the viability.

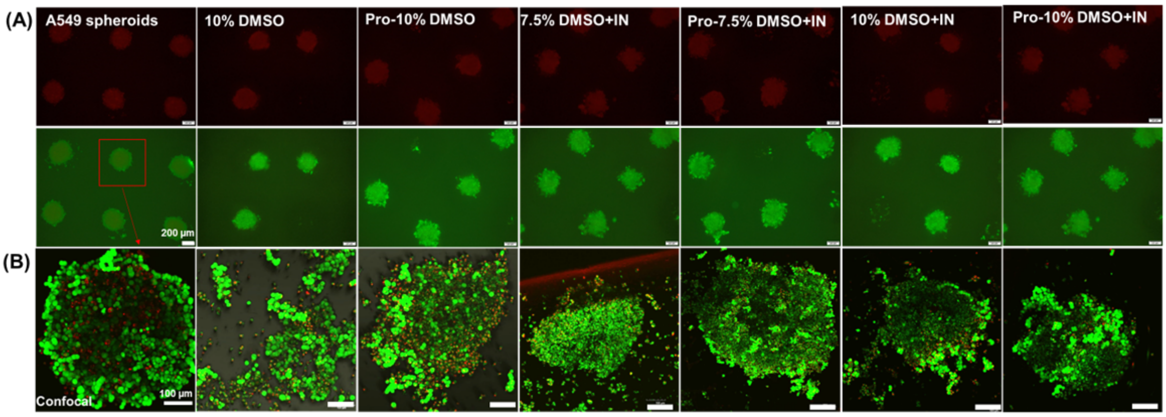

Figure S4. Morphology of spheroids before and after freezing. (A) Images captured by fluorescence microscope, cryopreserved using method B), and (B) confocal microscope, green (live cells, calcein-AM), red (dead cells, EthD-III). Pro: 300 mM L-proline pre-incubation, IN: ice nucleation agent (Pollen washing water). Scale bar: 200 µm (A), 100 µm (B).

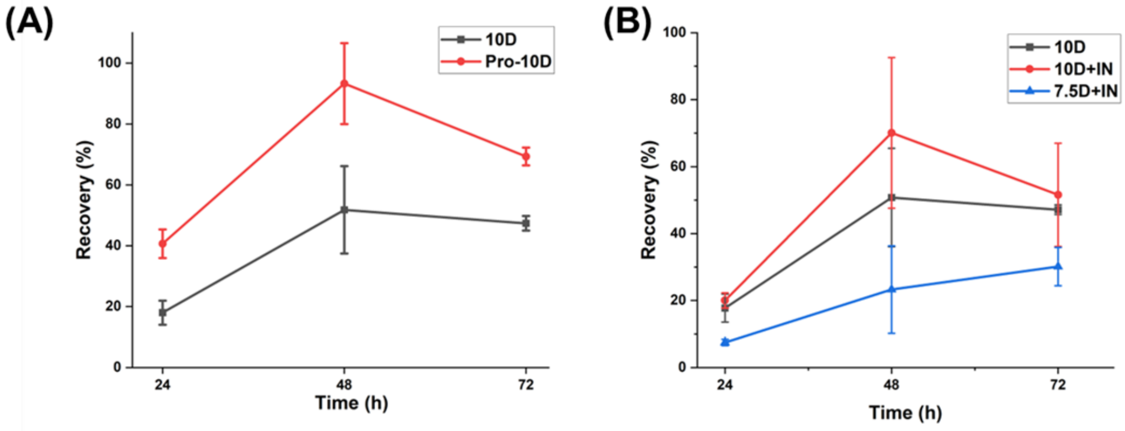

Figure S5. Recovery of A549 spheroids post-thaw 72 hr. (A) Percentage recovery of spheroids with or without 300 mM of L-proline preincubation for 24 h and cryopreserve with 10% DMSO, recovery of spheroids increased significantly after post-thaw 48 h with Pro preincubation. (B) Percentage recovery of spheroids with or without various concentrations of IN with 10% DMSO for cryopreservation, IN group improved recovery of spheroids after post-thaw 48 h compared to 10% DMSO alone group.

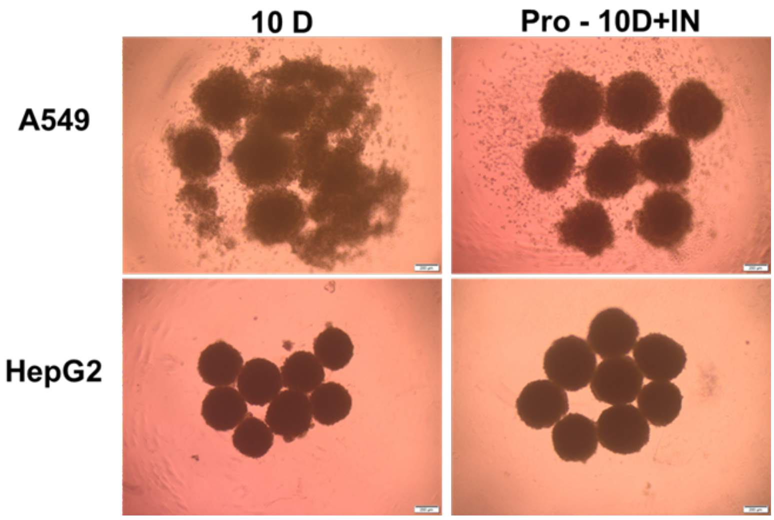

Figure S6. Post-thaw spheroids in cryovial. 8000 cells/spheroid, 8 spheroids/well in U-96 well plate after thawing and culture for 24 h.

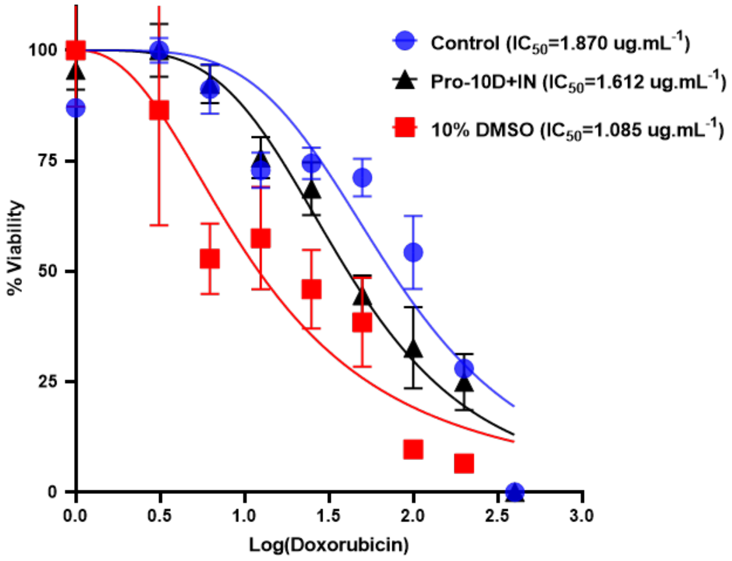

Figure S7. Cytotoxicity of HepG2 spheroids with Doxorubicin incubation for 24 hours. IC50 values indicated in the legend, CellTiter-Glo® 3D Cell Viability assay was used to determine the viability.

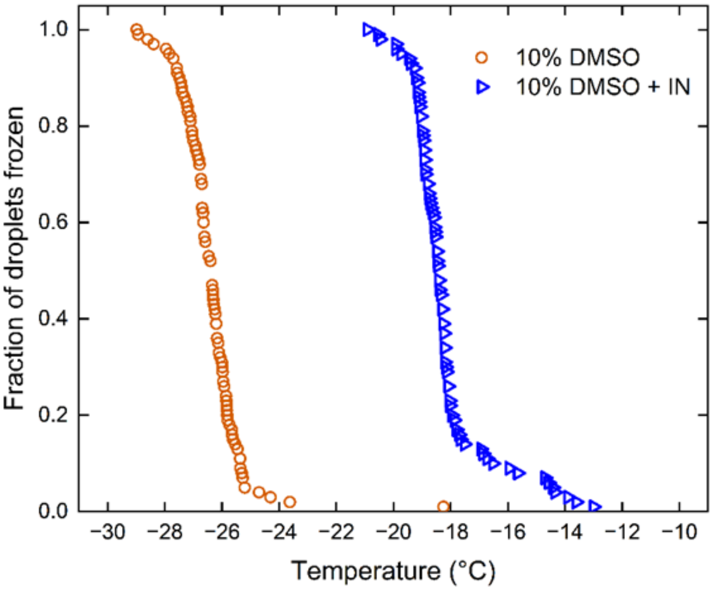

Figure S8. Freezing temperatures of 5 µl droplets of *Carpinus betulus* PWW made up in 10% DMSO (10% DMSO + IN) compared to 10% DMSO. 100 droplets of each solution were frozen.

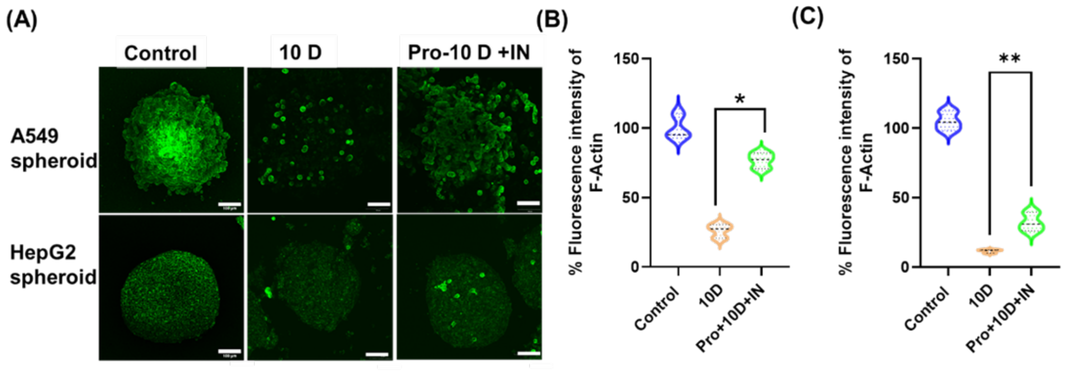

Figure S9. Effect of cryopreservation on cytoskeleton integrity. (A) Non-frozen control spheroids (left), spheroids cryopreserved in 10% DMSO (middle), preconditioning with 300 mM L-proline for 24 hours and cryopreserved in 10% DMSO inducing ice nucleation (right). The percentage fluorescence of F-actin of the cryopreserved samples (B) A549 spheroids (C) HepG2 spheroids, the data was plotted relative to the non-frozen control group. The scale bar is 100 µm (A). * P<0.05, ** P<0.01.
